# The association of regional block with intraoperative opioid consumption in patients undergoing video-assisted thoracoscopic surgery: a single-center, retrospective study

**DOI:** 10.1186/s13019-024-02611-3

**Published:** 2024-03-13

**Authors:** Yan Xiang, Liang Chen, Jiang Jia, Fu Yili, Wei Changwei

**Affiliations:** 1 Department of Anesthesiology, Beijing Chaoyang Hospital, Capital Medical University, No. 8 Gongren Tiyuchang Nanlu, Chaoyang District, Beijing, 100020 China; 2 Department of Medical Statistics, Medieco Group Co., Ltd, Beijing, China; 3 Department of Thoracic surgery, Beijing Chaoyang Hospital, Capital Medical University, Beijing, China

**Keywords:** Thoracic surgery, Video-assisted, Regional analgesia, Opioids

## Abstract

**Background:**

Regional block, such as thoracic epidural analgesia (TEA), thoracic paravertebral block (TPVB), or serratus anterior plane block (SAPB) has been recommended to reduce postoperative opioid use in recent guidelines, but the optimal options for intraoperative opioid minimization remain unclear. The aim of this study was to evaluate the intraoperative opioids-sparing effects of three regional blocks (TEA, TPVB, and SAPB) in patients undergoing video-assisted thoracoscopic surgery (VATs).

**Methods:**

This was a retrospective study of the adults undergoing VATs at a tertiary medical center between January 2020 and February 2022. According to the type of regional block used, patients were classified into 4 groups: GA group (general anesthesia without any regional block), TEA group (general anesthesia combined with TEA), TPVB group (general anesthesia combined with TPVB), and SAPB group (general anesthesia combined with SAPB). Cases were matched with a 1:1:1:1 ratio for analysis by age, sex, ASA physical status, and operation duration. The primary outcome was the total intraoperative opioid consumption standardized to Oral Morphine Equivalents (OME). Multivariable linear regression was used to estimate the association of the three regional blocks with the OME.

**Results:**

A total of 2159 cases met the eligibility criteria. After matching, 168 cases (42 in each group) were included in analysis. Compared with GA without any reginal block, the use of TEA, TPVB, and SAPB reduced the median of intraoperative OME by 78.45 mg (95% confidence interval [CI], -141.34 to -15.56; *P =* 0.014), 94.92 mg (95% CI, -154.48 to -35.36; *P =* 0.020), and 11.47 mg (95% CI, -72.07 to 49.14; *P =* 0.711), respectively.

**Conclusions:**

The use of TEA or TPVB was associated with an intraoperative opioid-sparing effect in adults undergoing VATs, whereas the intraoperative opioid-sparing effect of SAPB was not yet clear.

**Supplementary information:**

The online version contains supplementary material available at 10.1186/s13019-024-02611-3.

## Introduction

With the popularization of high resolution CT, more lung nodules and cancers are identified with the incidental screening or evaluation on lung disease [1]. Considering the high mortality of cancer worldwide [2, 3], surgical resection is still one of the main options for suspected malignant nodules [1, 4]. Due to the strong stimulation of thoracotomy, patients undergoing thoracic surgery generally received higher doses of opioids than those undergoing abdominal, urology, gynecology, head, or neck surgeries [5]. Minimal invasive video-assisted thoracoscopic surgery (VATs) reduces the pain of surgery to a certain extent, but still 38% of patients experience moderate to severe pain after VATs [6]. 

Excessive opioids for intraoperative analgesia will induce acute opioid tolerance and hyperalgesia [7], as well as postoperative hypoventilation, nausea and vomiting, constipation, and urinary retention, those complications can increase hospital stay by 3 days and overall costs by 27% [8–11]. For these reasons, minimization of opioid usage is a key pillar of enhanced recovery after surgery (ERAS) in patients undergoing VATs [2, 12, 13]. Previous studies have shown that compared patients without regional blocks, the thoracic epidural anesthesia (TEA), thoracic paravertebral block (TPVB), or serratus anterior plane block (SAPB) could reduce opioids consumption after VATs by depositing local anesthesia in the potential space of the epidural, paravertebral, or interfascial planes, respectively [11, 14, 15]. Several studies compared the perioperative opioid-sparing effect of TEA, TPVB, and SAPB in VATs, but the conclusions were uncertain due to the high heterogeneity of analgesic regimens among studies [5, 16, 17]. Therefore, more evidence for intraoperative opioids-sparing effect of the three regional blocks is needed.

The primary aim of this study was to evaluate the intraoperative opioid-sparing effect of TEA, TPVB, or SAPB, compared with controls during VATs. We hypothesized that general anesthesia combined with TEA, TPVB, or SAPB could reduce the opioids consumption in patients during VATs compared to general anesthesia without any regional block.

## Study design and participants

This study incorporated with a single-center retrospective cohort design. We extracted electronic anesthesia records of eligible patients between January 2020 and February 2022 at Beijing Chaoyang Hospital of Capital Medical University. Patients receiving VATs under general anesthesia with a bronchial intubation were included. A 3 cm and a 1 cm incision were made at the level of the anterior axillary line between the fourth and fifth ribs, and at the level of the midaxillary line between the eighth and ninth ribs, respectively. Those who was younger than 18 years old, experienced emergency surgery, surgery cancellation, intraoperative thoracotomy, severe intraoperative complications (ClassIntra grade > III) [18], or without intraoperative anesthesia information were excluded. Patients who received regional block after skin incision or other regional blocks than TEA, TPVB, or SAPB were also excluded from this study.

The study was approved by the ethics committee of Beijing Chao-Yang hospital with a waiver of written consent (NO. 2021 − 689) and performed in accordance with the Declaration of Helsinki and the STROBE guideline [19]. 

### Procedures and measurements

The demographic characteristics, surgical and anesthetic data, perioperative medications, post-anesthesia care unit (PACU) duration, and length of postoperative hospital stay were obtained from the electronic medical records and reviewed by researchers manually. Demographic characteristics included age, gender, body mass index, American Society of Anesthesiologists (ASA) physical status, benign or malignant tumor, and medical history. Surgical and anesthetic data referred to the type of surgery, professional title (consultant, associate consultant, registrar) of the surgeon and anesthesiologist with experience in regional blocks, volume of blood loss, duration of surgery (time from incision to end of suture) and anesthesia (time from anesthesia induction to extubation), method of the maintenance of general anesthesia (total intravenous anesthesia [TIVA] or balanced anesthesia), type of regional block combined. Intraoperative medications of interest included opioids, sedatives, muscle relaxants, nonsteroidal anti-inflammatory drugs (NSAIDs), glucocorticoids, and regional anesthetics used in the operating room. The clinical practices for opioid use at this facility are as follows: sufentanil 0.2–0.3 ug/kg during induction, followed by continuous intravenous infusion of remifentanil 0.1–0.2 ug/kg/min depending on the patient’s hemodynamic response to surgical stimulation. After surgery, a multimodal analgesia regimen was implemented for all patients and no opioids are administered intraoperatively through patient control analgesia. Patients received a continuous intravenous analgesia (sufentanil 1.5ug/kg in 100 ml, a rate of 2 ml/h) for postoperative analgesia; Patients with thoracic epidural puncture catheter received continuous epidural analgesia (sufentanil 1ug/kg and ropivacaine 200 mg in 200 ml, a rate of 6 ml/h) for postoperative analgesia. In addition, all patients received oral ibuprofen (0.2 g per 8 h) until 48 h. Rescue analgesic was given when patients needed. The time-to-first rescue analgesic within 72 h after the VATs, and postoperative cardiopulmonary-related complications in hospital were also collected from electronic medical records.

According to the type of regional block applied before incision, patients were classified into the following 4 groups, (1) GA group, in which patients received general anesthesia without any regional block; (2) TEA group, in which patients received general anesthesia combined with thoracic epidural analgesia; (3) TPVB group, in which patients received general anesthesia combined with a single thoracic paravertebral block; (4) SAPB group, in which patients received general anesthesia combined with a single serratus anterior plane block. After a preliminary analysis of the entire cohort, it was found that the cases receiving TEA (*n* = 265), TPVB (*n* = 222), and SAPB (*n* = 99) were significantly lower than in the GA group (*n* = 1252). Additionally, there were statistically significant differences in ASA physical status (*P* = 0.028) and surgery duration (*P* < 0.001) between the groups. Then, cases were matched among 4 groups in 1:1:1:1 ratio based on age (± 2 years), sex, ASA physical status, and duration of surgery (± 15 min) for analysis.

The primary outcome was the total opioids consumption (standardized as oral morphine equivalents [OME]) during VATs in operating room [20]. The opioids available in this institution were sufentanil, remifentanil, and tramadol.

Secondary outcomes included: (1) time-to-first rescue analgesic within 72 h after VATs. The rescue analgesics was defined as additional opioids or NSAIDs required; (2) PACU duration. Patients were admitted to the PACU after extubation and were discharged until the Aldrete score reached 10; [21] (3) length of postoperative hospital stay, defined as from end of surgery to discharge from hospital in days. (4) incidence of postoperative complications, defined as cardiopulmonary-related complications graded as I or more according to the Clavien-Dindo score [22] during hospitalization.

## Statistical methods

All variables were summarized by descriptive statistics. Continuous variables were expressed as medians (quartiles), and overall comparisons among groups were performed using the Kruskal-Wallis H test among groups. Categorical variables were expressed as the number of cases (percentage), and the overall comparison among groups was performed using the Chi-square test, if the count in a cell was < 5, then the Fisher’s exact test was used.

For the intraoperative OME, the overall difference among the 4 groups (GA, TEA, TPVB, and SAPB) was determined by Kruskal-Wallis H test. Multiple pairwise comparisons among groups were further performed, and the Bonferroni method was used for correction [23]. Finally, generalized linear regression was used to assess the opioid-sparing effect adjusting for age, sex, weight, TIVA, steroids, NSAIDs [5], and unbalanced variables (*P* < 0.1).

The time-to-first recue analgesic among 4 groups were presented by Kaplan-Meier curve, and overall statistical significance was analyzed using log-rank test. For secondary outcomes that were statistically different in the overall comparison, a further analysis was done by regression models adjusted for confounding factors and unbalanced variables (*P* < 0.1).

Power analysis showed that at least 26 patients in each group (no less than 104 patients in total) were required to have 90% power to identify the regional block group with a 20% mean reduction in intraoperative OME, assuming α = 0.05, the standard deviation of OME in the control group was 20% of the mean. To satisfy the requirement that the number of subjects for each variable in the regression analysis should not be less than 10, we included a sample size large enough to accommodate an additional sample in the multivariate regression model. Statistical analysis was performed using IBM SPSS 24.0 and *P* < 0.05 was considered statistically significant.

## Results

**Fig. 1:**
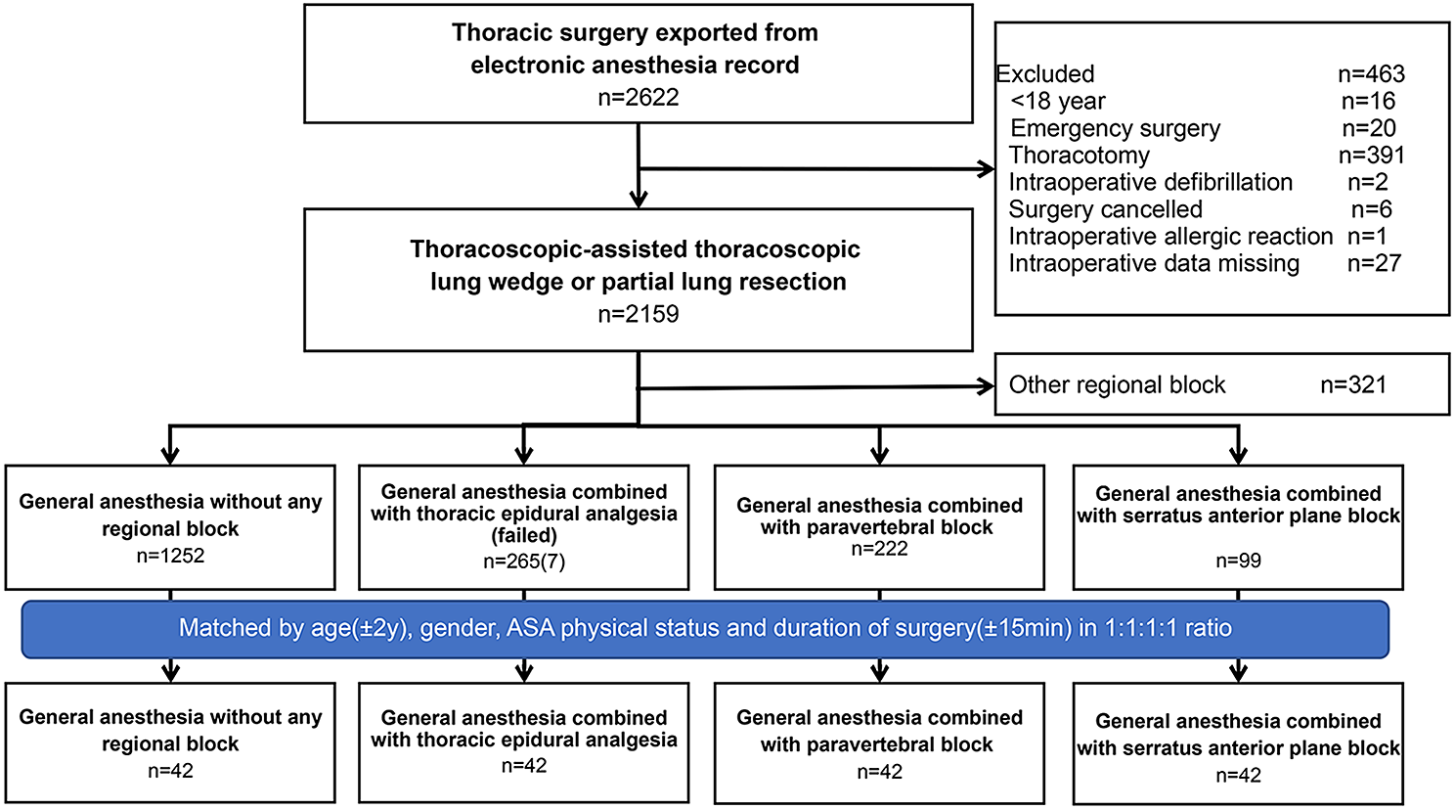
Study flowchart

A total of 2159 cases met the inclusion criteria. Among them, 1252 cases (57.9%) didn?t receive any regional block, 265 cases (12.3%) received TEA (7 cases failed), 222 cases (10.3%) received TPVB, and 99 cases (4.6%) received SAPB. Forty-two cases in each group were matched for analysis. See Figure 1. 

Of the 168 cases included in final analysis, the median [interquartile range] age was 61 [55, 66] years, 104 (61.9%) were female, most (85.7%) were ASA physical status II, 127 (75.6%) had malignant lung tumors. The demographic and surgical characteristics of the patients were comparable among the groups. See Tables 1 and 2. The surgical and anesthetic characteristics were comparable among the four groups except for the percentage of consultant anesthesiologist (14.3 vs. 42.9 vs. 14.3 vs. 0.0%, *P <* 0.001), percentage of TIVA (85.7 vs. 69.0 vs. 59.5 vs. 19.0%, *P <* 0.001), the dosage of propofol used during surgery (790 vs. 560 vs. 550 vs. 370 mg, *P <* 0.001), and the percentage of intraoperative flurbiprofen use (64.3 vs. 21.4 vs. 42.9 vs. 28.6%, *P <* 0.001). See Table 2.

**Table 1:** Demographics and baseline characteristics

	GA n = 42	TEA n = 42	TPVB n = 42	SAPB n = 42	*P* value
**Age, year**	62 [55, 65]	61 [55, 66]	61 [55, 66]	61 [54, 66]	> 0.999
**Gender, male**	16 (38.1)	16 (38.1)	16 (38.1)	16 (38.1)	> 0.999
**Height, cm**	162 [158, 170]	162 [156, 170]	165 [160, 171]	162 [156, 169]	0.461
**Weight, kg**	65 [60, 73]	66 [60, 72]	68 [60, 77]	68 [62, 72]	0.668
**BMI, kg/m** ^**2**^	24.7 [22.2, 27.1]	24.5 [22.1, 26.7]	25.0 [22.8, 27.3]	25.3 [23.7, 27.3]	0.508
**ASA physical status**					> 0.999
** II**	36 (85.7)	36 (85.7)	36 (85.7)	36 (85.7)	
** III**	6 (14.3)	6 (14.3)	6 (14.3)	6 (14.3)	
**Malignant tumor**	31 (73.8)	31 (73.8)	32 (76.2)	33 (78.6)	0.978
**Medical history**					
** Hypertension**	21 (50.0)	7 (16.7)	18 (42.9)	20 (47.6)	0.006*
** Diabetes**	8 (19.0)	4 (9.5)	6 (14.3)	7 (16.7)	0.706
** Coronary heart disease**	2 (4.8)	2 (4.8)	7 (16.7)	6 (14.3)	0.133
** Chronic obstructive pulmonary disease**	2 (4.8)	9 (21.4)	1 (2.4)	1 (2.4)	0.004*
** Stroke**	1 (2.4)	0 (0)	2 (4.8)	3 (7.1)	0.516
** Lung surgery**	0 (0)	0 (0)	0 (0)	1 (2.4)	> 0.999

Continuous variables were expressed as median [Interquartile range] and compared between groups will be performed using the Kruskal-Wallis H rank-sum test. Categorical variables were expressed as numbers (percentages), and comparisons between groups were performed using the chi-square test or Fisher’s exact test if a cell count was less than 5. *P < 0.05

Abbreviations: GA: General anesthesia without regional block; TEA: Thoracic epidural analgesia combined with general anesthesia; TBVP: Thoracic paravertebral block combined with general anesthesia; SABP: Serratus anterior plane block combined with general anesthesia; BMI: Body mass index; ASA: American Society of Anesthesiologists physical status classification

**Table 2:** Surgical and anesthetic characteristics

	GA n = 42	TEA n = 42	TPVB n = 42	SAPB n = 42	*P* value
**Type of lung surgery**					0.857
** Wedge resection**	10 (23.8)	10 (23.8)	7 (16.7)	10 (23.8)	
** Lobectomy**	32 (76.2)	32 (76.2)	35 (83.3)	32 (76.2)	
**Duration of surgery, min**	113 [94,130]	113 [85,130]	108 [85,135]	112 [89,130]	> 0.999
**Title of surgeon**					0.889
** Consultant**	35 (83.3)	36 (85.7)	35 (83.3)	33 (78.6)	
** Associate Consultant**	7 (16.7)	6 (14.3)	7 (16.7)	9 (21.4)	
**Blood loss, ml**	50 [20,50]	50 [30,100]	50 [20,50]	25 [20,50]	0.120
**Duration of anesthesia, min**	185 [170,220]	197 [170,215]	187 [160,220]	180 [162,205]	0.433
**Title of anesthesiologist**					< 0.001*
** Consultant**	6 (14.3)	18 (42.9)	6 (14.3)	0 (0)	
** Associate Consultant**	14 (33.3)	13 (31.0)	22 (52.4)	40 (95.2)	
** Registrar**	22 (52.4)	11 (26.2)	14 (33.3)	2 (4.8)	
**Maintenance of general anesthesia**					< 0.001*
** Total intravenous anesthesia**	36 (85.7)	29 (69.0)	25 (59.5)	8 (19.0)	
** Balanced anesthesia**	6 (14.3)	13 (31.0)	17 (40.5)	34 (81.0)	
** MAC of inhalation**	0.5 [0.4,0.5]	0.5 [0.5,0.5]	0.5 [0.5,0.5]	0.5 [0.5,0.6]	0.460
**Intraoprative medication administration**					
** Propofol, mg**	790 [600,900]	560 [370,750]	550 [350,720]	370 [260,550]	< 0.001*
** Rocuronium, mg**	70 [60,80]	65 [60,75]	70 [50,75]	65 [50,70]	0.483
** Midazolam**	28(66.7)	21(50.0)	22(52.4)	21(50.0)	0.382
** Midazolam, mg**	1 [0,1.5]	0.5 [0,2.0]	1.0 [0,1.0]	0.5 [0,1.0]	0.522
** Dexamethasone**	22(52.4)	23(54.8)	25(59.5)	25(59.5)	0.910
** Dexamethasone, mg**	5[0,10]	5[0,10]	5[0,10]	5[0,10]	0.906
** Flubiprofen**	27(64.3)	9(21.4)	24(42.9)	12(28.6)	< 0.001*
** Flubiprofen, mg**	50 [0,100]	0 [0,0]	0 [0,50]	0 [0,50]	< 0.001*
** Regional block anesthetics**					
** Volume of regional anesthetics, ml**	/	9 [7,13]	20 [20,20]	33 [30,40]	< 0.001*
** Ropivacaine**	/	17 (40.5)	42 (100)	42 (100)	< 0.001*
** Ropivacaine concentration, %**	/	0.43 [0.3,0.5]	0.5 [0.5,0.5]	0.32 [0.25,0.33]	< 0.001*
** Lidocaine**	/	25 (59.5)	0 (0)	0 (0)	
** Lidocaine concentration, %**	/	2 [2,2]	/	/	

Continuous variables were expressed as the median [Interquartile range] and compared between groups will be performed using the Kruskal-Wallis H rank-sum test. Categorical variables were expressed as numbers (percentages), and comparisons between groups were performed using the chi-square test or Fisher’s exact test if a cell count was less than 5. *P < 0.05

Abbreviations: GA: General anesthesia without regional block; TEA: Thoracic epidural analgesia combined with general anesthesia; TBVP: Thoracic paravertebral block combined with general anesthesia ; SABP: Serratus anterior plane block combined with general anesthesia; MAC: minimum alveolar concentration

The primary outcome, the total intraoperative OME, was significantly different among the four groups (median: 512 vs. 411 vs. 384 vs. 461 mg; *P* = 0.001). Since we further performed 6 pairwise comparisons between groups, the *P* value was adjusted by Bonferroni correction. Compared with the GA group, the median OME was lower in the TEA group (512 vs. 411 mg, Bonferroni adjusted *P =* 0.022) and TPVB group (512 vs. 384 mg; Bonferroni adjusted *P <* 0.001). There was no significant difference in the median OME between SAPB and GA group (512 vs. 461 mg; Bonferroni adjusted *P =* 0.168). See Table 3.

**Table 3:** Primary and secondary outcomes

	GA n = 42	TEA n = 42	TPVB n = 42	SAPB n = 42	*P* value
**Intraoperative OME, mg**	512 [450,630]	411 [360,540]*	384 [300,489]*	461 [381,540]	0.001
** Sufentanil IV, ug**	25 [20,30]	20 [20,30]	20 [15,30]	15 [10,20]	< 0.001
** Remifentanil IV, ug**	1420 [1200,1800]	1180 [920,1600]	1080 [800,1400]	1360 [1120,1600]	0.002
** Tramadol IV, mg**	0 [0,100]	0 [0,0]	0 [0,0]	0 [0,0]	< 0.001
**Rescue analgesic during the first 72 h after surgery**	18 (42.9)	15 (35.7)	12 (28.6)	7 (16.7)	0.069
** Time-to-first rescue analgesic after surgery, h**	18 [12,23]	16 [5,22]	18 [12,21]	18 [17,20]	0.718
**Postoperative complications**	1 (2.4)	4 (9.5)	5 (11.9)	0 (0)	0.154
** Pneumonia**	0 (0)	1 (2.4)	0 (0)	0 (0)	
** Pneumothorax**	0 (0)	1 (2.4)	2 (4.8)	0 (0)	
** Chylothorax**	0 (0)	1 (2.4)	2 (4.8)	0 (0)	
** Pleural effusion**	1 (2.4)	1 (2.4)	0 (0)	0 (0)	
** Atrial fibrillation**	0 (0)	0	1 (2.4)	0 (0)	
**PACU duration, min**	45 [35,60]	34 [28,45]	30 [15,45]	44 [29,59]	0.007
**Length of postoperative hospital stay**	5 [4,5]	4 [4,6]	4 [3,5]	3 [3,4]	< 0.001

Continuous variables were expressed as the median [Interquartile range] and compared between groups will be performed using the Kruskal-Wallis H rank-sum test. Categorical variables were expressed as numbers (percentages), and comparisons between groups were performed using the chi-square test or Fisher’s exact test if a cell count was less than 5.*Bonferroni adjusted *P* < 0.05

Abbreviations: GA: General anesthesia withou regional block; TEA: Thoracic epidural analgesia combined with general anesthesia; TBVP: Thoracic paravertebral block combined with general anesthesia; SABP: Serratus anterior plane block combined with general anesthesia; OME: Oral Morphine Equivalents; PACU: Post Anesthesia Care Unit; NSAIDs: Non-Steroidal Anti-Inflammatory Drugs; PONV: Postoperative Nausea and Vomiting.

Multivariable analysis identified the adjusted opioid-sparing effect of regional blocks. See Table 4. Compared with GA, TEA reduced intraoperative OME by 78.45 mg (95%CI, -141.34 to -15.56; *P =* 0.014), TPVB reduced intraoperative OME by 94.92 mg (95%CI, -154.48 to -35.36; *P =* 0.020), while SAPB group did not significantly reduce intraoperative OME (-11.47 mg, 95CI%, -72.07 to 49.14; *P =* 0.711)

**Table 4:** The association between type of regional block and intraoperative OME: generalized linear regression

Variable	Mean difference of OME in mg (95% CI)	adjusted *P* value
Type of regional block		
** GA**	Reference	-
** TEA**	-78.45 (-141.34 to -15.56)	0.014*
** TPVB**	-94.92 (154.48 to -35.36)	0.020*
** SAPB**	-11.47 (-72.07 to 49.14)	0.711
**Age per year increase**	2.17 (-0.54 to 4.88)	0.117
**Female sex (yes vs. no)**	-14.76 (-62.83 to 33.30)	0.547
**Weight per kg increase**	3.11 (0.63 to 5.58)	0.014*
**Chronic obstructive pulmonary disease ** **(yes vs. no)**	39.76 (-39.12 to 118.64)	0.323
**Hypertension (yes vs. no)**	-36.32 (-76.93 to 4.30)	0.080
**Total intravenous anesthesia ** **(yes vs. no)**	65.50 (13.68 to 117.33)	0.013*
**Flubiprofen per mg increase**	0.21 (-0.37 to 0.78)	0.480
**Dexamethasone per mg increase**	-2.25 (-6.50 to 2.00)	0.299
**Title of anesthesiologist**		
** Registrar**	Reference	-
** Associate Consultant**	-0.34 (-53.86 to 53.18)	0.990
** Consultant**	23.04 (-55.27 to 101.34)	0.564

These confounders were all entered into a generalized linear regression including: age, sex, weight, TIVA, dexamethasone, flubiprofen, and variables with *P* < 0.1 in univariate analysis (chronic obstructive pulmonary disease, hypertension, title of anesthesiologist). *N* = 168. *adjusted *P* < 0.05

Abbreviations: OME: oral morphine equivalents; CI: confidence interval; GA: General anesthesia without regional block; TEA: Thoracic epidural analgesia combined with general anesthesia; TBVP: Thoracic paravertebral block combined with general anesthesia; SABP: Serratus anterior plane block combined with general anesthesia

There was no overall significant difference in the time-to-first rescue analgesia within 72 h after VATs among the 4 groups (*P =* 0.065). See Supplementary Fig. 1. Compared with the GA group, the median PACU duration in TPVB was decreased by 14.71 min (95% CI, -23.18 to -6.25; *P =* 0.001), while the use of TEA or SAPB had no significant effect on PACU duration in multivariate analysis. See Supplementary Table (1) Compared with GA, the length of postoperative hospital stay in the SAPB group was decreased by 1.28 days (95% CI, -2.21 to -0.35, *P =* 0.007). See Supplementary Table (2) No significant difference was observed in the incidence of postoperative complications among the groups. See Table 3.

## Disscussion

This retrospective study showed that compared with GA, the use of TEA or TPVB was associated with an intraoperative opioid-sparing effect in patients undergoing VATs. While no significant intraoperative opioid-sparing effect was observed with SAPB.

We observed that compared with GA, TEA reduced the median intraoperative OME by 15.3%, which was lower than those in previous studies (27%$\sim$46%) [11, 24]. The reason may be that more patients in our study underwent a less invasive wedge resection, and we did not give any opioids through thoracic epidural catheter intraoperatively. Ultrasound-guided regional block techniques, such as TPVB and SAPB, have been popularized for the high success rate and good safety in postoperative analgesia after VATs [13, 25]. Our results showed that compared with GA, TPVB reduced the median intraoperative OME by 18.5%. The intraoperative opioid-sparing effect of TPVB was comparable to that of TEA in VATs, this is consistent with the results of some randomized controlled studies [26, 27]. SAPB, a novel regional block of interest, was shown to be noninferior to TPVB for 48 h postoperative opioids consumption after VATs [25, 28]. Our data did not observe a significant intraoperative opioid-sparing effect of SAPB in patients undergoing VATs. Similarly, a meta-analysis included 171 patients undergoing VATs also didn’t find a significant intraoperative opioid sparing-effect of SAPB [16]. This may be due to the inability of SAPB to modulate the nociceptive triggers generated by deep visceral stimulation during surgery, and the analgesic effect of SAPB requires the diffusion of local anesthetic in the fascia space over a period of time [16]. Recent network meta-analysis compared the analgesic effect of TEA, TPVB, SAPB, erector spinae plane block (ESPB), and intercostal nerve block, and concluded that TEA, TPVB, and ESPB had better analgesia then others [29]. 

TEA has been the gold standard for postoperative analgesia after thoracic surgery for decades [12, 13]. Current guidelines more recommend TPVB as an alternative to TEA for the following reasons [26, 30]. First, the analgesic effect (assessed by the pain score at rest or coughing within 48 h after surgery) of TPVB is comparable to that of TEA in patients under multimodal analgesia regimens after VATs [17, 31]. Second, TEA delivers the anesthetic to the epidural space, while TPVB delivers the anesthetic only to the unilateral thoracic paravertebral space with less impact on spinal cord function. This may explain why TPVB showed a lower side-effect profile than TEA, including hypotension, nausea and vomiting, pruritis, and urinary retention after thoracotomy [10, 11, 17, 31, 32]. In addition, due to the extreme caudal angulation of the thoracic spinous processes [11], the TEA is tough and time-consuming with potential risks of arachnoid puncture and spinal cord injury. While, with the introduction of ultrasound, the TPVB becomes simple, and the failure rate as well as the risk of pneumothorax have been reduced [31]. 

An excellent postoperative pain management is important for early postoperative mobility, reduce postoperative complications, and shorten hospital stay [12, 13]. Regional blocks were believed to be potential to promote early mobilization in patients after VATs since it could reduce the incidence of moderate to severe pain in the early postoperative period [10, 11, 28, 33]. It was reported that the use of regional block reduced postoperative opioid consumption and prolonged the time-to-first rescue analgesic in patients after VATs compared with those who did not receive regional blocks [14, 24, 33, 34]. Recent studies compared the analgesic effects of TEA, TPVB, and SAPB in patients after VATs and found that they were comparable in terms of opioid-sparing effects and postoperative pain scores [17, 24, 25, 28, 30, 32]. Our study did not show that the choice of regional block was associated with prolonged time-to-first rescue analgesic. The reason may be that with an effective multimodal analgesia for VATs, the difference in the time-to-first rescue analgesic among groups was not significant. We did not observe the postoperative pain scores or opioids consumption due to incomplete medical records and a continuous analgesic pump without patient control used after surgery. We observed that compared with GA, TPVB shortened the median PACU duration by 15 min, while SAPB reduced median length of hospital stay by 1 day. Er, et al. reported that compared with GA, SAPB was associated with shorter hospital stay, as it was associated with improved quality of recovery scores by reducing early postoperative pain scores [33]. Nevertheless, the reduction in length of PACU or hospital stay observed in our study did not seem to be encouraging enough for clinical practice. As to the postoperative complications, we could not draw any conclusion due to small sample size.

For further opioid reduction, an opioid-free anesthesia regimens consisting of regional blocks combined with non-opioid analgesics, have been successful applied in patients undergoing VATs recently [35, 36]. The novel regimens showed a comparable pain control intraoperatively (assessed by electroencephalogram) [36] and lower opioids consumption postoperatively compared with standard anesthesia [35]. It is still unclear that whether there will be a relationship between opioid-free anesthesia and early recovery quality in patients after VATs [35, 36], further well-designed randomized controlled trails are warranted.

To our knowledge, this is the first study to evaluate the intraoperative opioid-sparing effect of TEA, TPVB, and SAPB under multimodal analgesia regimens in patients undergoing VATs. However, this study has several limitations. First, to ensure that there were no major changes in clinical practice, only two years’ data in a single institution were extracted. Although this might result in small sample size, the available cases collected still met the minimum requirements for testing the primary outcome. Second, anesthesiologists might have preferences in implementing analgesic regimens, and the subtle heterogeneity among practitioners still inevitable [17]. Therefore, we collected the title of anesthesiologists for analysis to make the conclusions relatively generally applicable. Third, the main purpose of multimodal analgesia is to minimize opioid use and promote early recovery in patients undergoing VATs. However, due to the property of the retrospective study, the choice of rescue analgesics and the time of discharge may be subjectively determined by the surgeons, which would bias the results. For the same reason, although we matched patients for important factors and further adjusted for imbalance factors to verify the robustness of the conclusion, some unknown risk factors could not be adjusted. Finally, the intraoperative opioid administration was determined by the discretion of the anesthesiologist according to hemodynamic parameters, this may provide the benefit of eliminating the Hawthorne effect for the opioid consumption during operation.

## Conclusion

Our study demonstrates that compared with GA, the use of TEA or TPVB provide an opioid-sparing effect in adult patients undergoing VATs, whereas the opioid-sparing effect of SAPB is not yet clear. Regional blocks have the potential to facilitate postoperative recovery time both in PACU or discharge, albeit this benefit may not be clinically significant in our study. Our findings in concert with recent guideline suggest that the use of regional block reduced opioid consumption intraoperatively, and should be considered as part of an optimal analgesic strategy for patients undergoing VATs. Further randomized control trial is required to explore the optimal regional-block based multimodal analgesia regimens for opioid-free anesthesia in patients undergoing VATs.

### Electronic supplementary material

Below is the link to the electronic supplementary material.

Supplementary Material 1

Supplementary Material 2

Supplementary Material 3

## Data Availability

The datasets used and/or analyzed during the current study available from the corresponding author on reasonable request.

## Abbreviations

TEA: Thoracic Epidural Analgesia
TPVB: Thoracic Paravertebral Block
SAPB: Serratus Anterior Plane Block
VATs: Video-Assisted Thoracoscopic Surgery
GA: General Anesthesia
OME: Oral Morphine Equivalents
CI: Confidence Interval
ERAS: Enhanced Recovery After Surgery
PACU: Post-Anesthesia Care Unit
ASA: American Society of Anesthesiologists
TIVA: Total Intravenous Anesthesia
NSAIDs: Nonsteroidal Anti-Inflammatory Drugs
ESPB: Erector Spinae Plane Block
