# Supplementary material for: The association of regional block with intraoperative opioid consumption in patients undergoing video-assisted thoracoscopic surgery: a single-center, retrospective study
Source: J Cardiothorac Surg. 2024 Mar 13;19:124. doi: 10.1186/s13019-024-02611-3 (PMC10936020; doi:10.1186/s13019-024-02611-3)
Supplement: Supplementary file 1 — Supplementary Material 1 [file 13019_2024_2611_MOESM1_ESM.docx]

**Supplementary Figure 1. The Kaplan-Meier curve of the first rescue analgesic in patients receiving different regional block within 72 hours after surgery.
GA, General anesthesia; TEA, Thoracic epidural analgesia; TPVB, Thoracic paravertebral block; SABP, Serratus anterior plane block.**


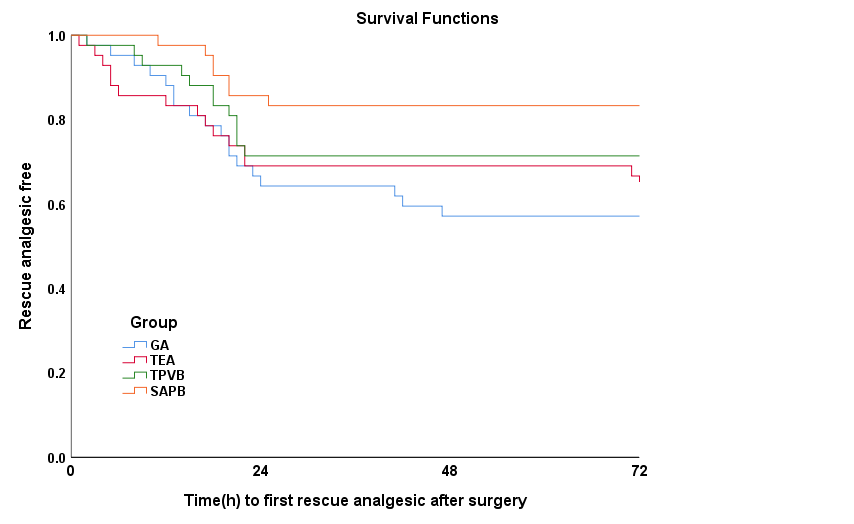


Log-rank *P*-value= 0.065
