# Supplementary material for: The association of regional block with intraoperative opioid consumption in patients undergoing video-assisted thoracoscopic surgery: a single-center, retrospective study
Source: J Cardiothorac Surg. 2024 Mar 13;19:124. doi: 10.1186/s13019-024-02611-3 (PMC10936020; doi:10.1186/s13019-024-02611-3)
Supplement: Supplementary file 2 — Supplementary Material 2 [file 13019_2024_2611_MOESM2_ESM.docx]

| **Supplementary TABLE 1. The association between Type of regional block and Length of PACU Duration: Generalized linear regression** | | | |
| --- | --- | --- | --- |
| **Variable** | **Mean difference of PACU duration in min (95% CI)** | | **adjusted *P* value** |
| **Type of regional block** |  |  |  |
| **GA** | Reference | |  |
| **TEA** | -7.71 (-18.403 to 2.977) | | 0.157 |
| **TPVB** | -14.71 (-23.175 to -6.251) | | 0.001* |
| **SAPB** | -9.75 (-19.687 to 0.186) | | 0.054 |
| **Age per year increase** | 0.50 (0.066 to 0.929) | | 0.024***** |
| **Female sex (yes vs no)** | 1.62 (-5.098 to 8.343) | | 0.636 |
| **Body Mass Index per 1 kg/m^2^ increase** | 0.24 (-0.923 to 1.395) | | 0.690 |
| **Hypertension (yes vs no)** | 2.79 (-4.343 to 9.919) | | 0.443 |
| **Chronic obstructive pulmonary disease (yes vs no)** | 0.03 (-14.941 to 15.004) | | 0.997 |
| **Duration of surgery per min increase** | -0.01 (-0.128 to 0.100) | | 0.807 |
| **Total intravenous anesthesia** | 3.24 (-8.875 to 15.361) | | 0.600 |
| **Propofol per mg increase** | -0.01 (-0.031 to 0.018) | | 0.596 |
| **Midazolam per mg increase** | 4.18 (-0.458 to 8.824) | | 0.077 |
| **Rocuronium per mg increase** | 0.04 (-0.233 to 0.308) | | 0.787 |
| **Dexamethasone per mg increase** | 0.21 (-0.501 to 0.920) | | 0.563 |
| **Title of anesthesiologist** |  | |  |
| **Registrar** | Reference | |  |
| **Associate Consultant** | 6.70 (-2.964 to 16.366) | | 0.174 |
| **Consultant** | 3.05 (-7.198 to 13.303) | | 0.559 |
| These confounders with *P* <0.1 in univariate analysis were entered into a generalized linear regression including; N = 140. *adjusted *P* <0.05. Abbreviations: PACU: Post-anesthesia care unit; GA: General anesthesia without regional block; TEA: Thoracic epidural analgesia combined with general anesthesia; TBVP: Thoracic paravertebral block combined with general anesthesia; SABP: Serratus anterior plane block combined with general anesthesia. | | | |
