# Supplementary material for: The association of regional block with intraoperative opioid consumption in patients undergoing video-assisted thoracoscopic surgery: a single-center, retrospective study
Source: J Cardiothorac Surg. 2024 Mar 13;19:124. doi: 10.1186/s13019-024-02611-3 (PMC10936020; doi:10.1186/s13019-024-02611-3)
Supplement: Supplementary file 3 — Supplementary Material 3 [file 13019_2024_2611_MOESM3_ESM.docx]

| **Supplementary TABLE 2. The association between Type of regional block and Length of Postoperative Hospital Stay: Generalized linear regression** | | | |
| --- | --- | --- | --- |
| **Variable** | **Mean difference of PACU duration in day (95%CI)** | | **adjusted *P* value** |
| **Type of regional block** |  |  |  |
| **GA** | Reference | | - |
| **TEA** | -0.54 （-1.429 to 0.356) | | 0.239 |
| **TPVB** | -0.60 （-1.386 to 0.181) | | 0.132 |
| **SAPB** | -1.28 （-2.215 to -0.348) | | 0.007***** |
| **Age per year increase** | -0.03 (-0.091 to 0.027) | | 0.288 |
| **Female sex (yes vs no)** | 0.30 (-0.532 to 1.122) | | 0.485 |
| **Body mass index per 1 kg/m^2^ increase** | 0.06 (-0.020 to 0.137) | | 0.142 |
| **Hypertension (yes vs no)** | 0.18 (-0.694 to 1.046) | | 0.692 |
| **Chronic obstructive pulmonary disease (yes vs no)** | 0.86 (-0.103 to 1.818) | | 0.080 |
| **ASA III to II** | -0.16 (-0.993 to 0.671) | | 0.705 |
| **Malignant tumor (yes vs no)** | -0.47 (-1.608 to 0.671) | | 0.420 |
| **Wedge resection (yes vs no)** | -0.82 (-1.859 to 0.220) | | 0.122 |
| **Duration of surgery per 1 h increase** | 0.65 (0.065 to 1.225) | | 0.029* |
| **intraoperative OME per mg increase** | 0.00 (-0.003 to 0.005) | | 0.997 |
| **Total intravenous anesthesia (yes vs no)** | 0.69 (-0.363 to 1.735) | | 0.200 |
| **Title of anesthesiologist** |  | |  |
| **Registrar** | Reference | | - |
| **Associate Consultant** | 0.96 (0.009 to 1.902) | | 0.048***** |
| **Consultant** | -0.19 (-1.207 to 0.821) | | 0.709 |
| **Postoperative complications (yes vs no)** | 9.87 （5.562 to 14.167) | | <0.001***** |
| These confounders with *P* <0.1 in univariate analysis were entered into a generalized linear regression including; N = 168. *adjusted *P* <0.05. Abbreviations: GA: General anesthesia without regional block; TEA: Thoracic epidural block combined with general analgesia; TBVP: Thoracic paravertebral block combined with general anesthesia; SABP: Serratus anterior plane block combined with general anesthesia; OME: oral morphine equivalents. | | | |
